# Characterization and Genomic Analysis of Bacteriophage vB_KpnM_IME346 Targeting Clinical *Klebsiella pneumoniae* Strain of the K63 Capsular Type

**DOI:** 10.1007/s00284-022-02834-4

**Published:** 2022-04-13

**Authors:** Mingming Gao, Lingxian Yi, Yuan Wang, Jie Gao, Huiying Liu, Xianglilan Zhang, Guangqian Pei, Yigang Tong, Changqing Bai

**Affiliations:** 1 Department of Critical Care Medicine, PLA Strategic Support Characteristic Medical Center, Beijing, 100101 China; 2 Department of Respiratory and Critical Care Diseases, The Fifth Medical Center, Chinese General Hospital of the PLA, Beijing, 100071 China; 3 State Key Laboratory of Pathogen and Biosecurity, Beijing Institute of Microbiology and Epidemiology, Beijing, 100071 China; 4 Beijing Advanced Innovation Center for Soft Matter Science and Engineering (BAIC-SM), College of Life Science and Technology, Beijing University of Chemical Technology, Beijing, 100029 China

## Abstract

A *Klebsiella pneumoniae* bacteriophage (vB_KpnM_IME346) was isolated from a hospital sewage sample. This bacteriophage specifically infects a clinical *K. pneumoniae* strain with a K63 capsular polysaccharide structure. The phage genome was evaluated by next-generation sequencing, which revealed a linear double-stranded DNA genome consisting of 49,482 base pairs with a G+C content of 49.1%. The latent period of vB_KpnM_IME346 was shown to be 20 min, and the burst size was 25–30 pfu (plaque-forming units)/infected cell. Transmission electron microscopy and phylogenetic analysis showed that the JD001-like phage belongs to the genus *Jedunavirus* of the family *Myoviridae*. The newly isolated vB_KpnM_IME346 shows infectivity in the clinical host *K. pneumoniae* KP576 strain, indicating that it is a promising alternative to antibacterial agents for removing *K. pneumoniae* from patients.

**Supplementary Information:**

The online version contains supplementary material available at 10.1007/s00284-022-02834-4.

## Introduction

*Klebsiella pneumoniae* is a Gram-negative opportunistic pathogenic bacterium and common cause of hospital- and community-acquired urinary tract infections, sepsis, and pneumonia [1]. In recent decades, cases of community-acquired suppurative liver abscess, caused by *K. pneumoniae* and complicated with metastatic meningitis and endophthalmitis have emerged worldwide, particularly in Asia [2–4], and the clinical picture is rapidly progressing. Uncontrolled application of various antibacterial agents and frequent exposure of various *K. pneumoniae* isolates to antibacterial agents cause multidrug resistance among the strains [5]. Although antibiotics remain as the first-line treatment for *K. pneumoniae* infections, alternative treatments are urgently needed because of increasing rates of antibiotic resistance. Infections due to carbapenemase-producing *K. pneumoniae* (CPKp) have been recognized as an emerging challenge worldwide [6]. One possible strategy for effectively treating *K. pneumoniae* infections without risking drug-resistant strain development is to exploit the ability of lytic bacteriophages to target pathogenic bacteria.

Bacteriophages, also known as phages, are viruses that specifically recognize their bacterial hosts. Since their discovery 100 years ago phage research has changed basic biology and medicine. With increases in antibiotic resistance, phage therapy has provided a new perspective for treating infections [7]. Recently, an open study using a cocktail of 12 phages to treat patients with burn wound infections of *Pseudomonas aeruginosa* demonstrated the effectiveness of topical administration of phage therapy. This was the first clinical trial of phage therapy performed in compliance with both good manufacturing practices and good clinical practices [8]. Thus, phage therapy shows potential for clinical treatment. To date, approximately 100 Klebsiella phages have been isolated from different geographic regions, and the genomes of at least 30 of these phages have been deposited into the NCBI database. An essential virulence factor and a defense barrier of *K. pneumoniae* are a polysaccharide capsule (CPS). Bacteriophage-encoded polysaccharide-degrading enzymes are considered as effective tools for controlling bacteria covered with polysaccharide capsules. Majkowska-Skrobek et al. [9] and Volozhantsev et al. [10] used depolymerase as a tool to develop anti-virulent strategies.

In the present study, the previously unidentified bacteriophage vB_KpnM_IME346, which infects the *K. pneumoniae* clinical KP576 strain, a K63 capsular type [11], and its morphology, growth parameters, and genome were investigated.

## Materials and Methods

### Bacteria and Strain

*Klebsiella pneumoniae* KP576 strain was isolated from a patient at the Fifth Medical Center of the Chinese General Hospital of Chinese People’s Liberation Army in Beijing, China (The Fifth Medical Center of PLA). We previously identified *K. pneumoniae* strain KP576 as belonging to the K63 capsular type, as described by Pan [11]. The strain was cultured in Luria–Bertani (LB) medium at 37 °C.

### Phage Isolation

The virulent phage vB_KpnM_IME346 was isolated using KP576 as the indicator strain from sewage collected at the Fifth Medical Center of PLA. The phage was isolated according to previously described procedures [12] with minor modifications. Briefly, a sewage sample was obtained from the hospital and centrifuged at 10,000×*g* for 10 min. The supernatant was filtered through a 0.22-μm microporous membrane. A 3-mL aliquot of the filtrate was mixed with 500 μL of an exponentially growing *K. pneumoniae* KP576 LB culture (*OD*_600_ = 0.6) and 3 mL 3 × LB. This mixture was incubated overnight at 37 °C with shaking, followed by filtration through a 0.22-μm membrane. The double-layer agar procedure of phage isolation was repeated four times.

### Biological Properties

A one-step growth experiment was performed to determine the lysis curve and phage burst size, as described previously [13]. Briefly, the phage and bacterial culture in the log phase were mixed at a multiplicity of infection of 0.01 (10^7^:10^9^ number of phage/number of bacteria ratio), and the phage was allowed to adsorb to the bacterial cells for 1 min at 37 °C. The mixture was washed with LB medium to remove unabsorbed phages and avoid secondary adsorption. The culture was incubated at 37 °C with shaking, and samples were collected at 5- and 10-min intervals. The phage titers were then determined using the double-layer agar method. Phage morphology was further visualized and characterized by transmission electron microscopy (JEM-1200EX, Jeol, Tokyo, Japan) at an accelerating voltage of 100 kV.

### Host Range Determination

Bacterial strain susceptibility levels were determined as previously described [12]. A total of 12 *K. pneumoniae* (including capsular type K63 (3/12), K47 (4/12), KN3 (1/12), K64 (2/12), and K81 (2/12)) strains were used for host range evaluation. First, the spotting method was used to evaluate the susceptibility of the bacterial strain to the phage and then the efficiency of plating was determined [14] by the double-layer agar method. The efficiency of plating values was determined by calculating the ratio of plaque-forming units of each phage-susceptible strain to plaque-forming units of the indicator strain (*K. pneumoniae* KP576). This experiment was repeated three times.

### DNA Extraction, Whole-Genome Sequencing, and Genomic Analysis

The genomic DNA of the phage was extracted using a standard phenol–chloroform protocol [12]. Briefly, the purified phages were treated by proteinase K (100 mg/mL), SDS (10%, w/v), and EDTA (0.5 mM, pH 8.0) at 56 °C in water for 2 h. After that, the sample was washed three times using an equal volume of mixture composed of phenol, chloroform, and isoamyl alcohol (25:24:1), followed by centrifugation at 4 C, 12,000×*g* for 10 min, to remove the debris. Then, the supernatant was mixed with isoamyl alcohol kept at 20 °C overnight. The air-dried precipitate was washed three times with cold 75% ethanol, and the phages’ genomic DNA was finally dissolved in TE buffer (10 mM Tris–HCl, 1 mM EDTA [pH 8.0]).

High-throughput sequencing of the phage genomic DNA was performed using the Illumina MiSeq platform (San Diego, CA, USA). The whole-genome sequence was assembled using Newbler V3.0 software [15], and annotations were conducted using the RAST online database (http://www.rast.nmpdr.org) [16]. Antimicrobial resistance and bacterial virulence factors were determined from the following databases: Virulence Factor Database [17], Comprehensive Antibiotic Resistance Database [18], Antibiotic Resistance Gene-ANNOTation [19], and ResFinder [20]. Transfer RNAs (tRNAs) were predicted using the tRNAscan-SE software [21]. To identify gene homologs, the genomes of vB_KpnM_IME346 and vB_KpnM_KpV52 were analyzed in CoreGenes3.5 for protein identity (http://binf.gmu.edu:8080/CoreGenes3.5/). The DNA sequence of the polymerase-encoding gene of phage vB_KpnM_IME346 and other homologous sequences, obtained from the International Committee on Taxonomy of Viruses (https://talk.ictvonline.org/taxonomy/), was used to construct a phylogenetic tree using MEGA 6.0 [22] and neighbor-joining method using 1000 guide repeats. All analyses except those indicated were performed using default parameters.

## Results and Discussion

### Phage Isolation and Morphology

**Fig. 1:**
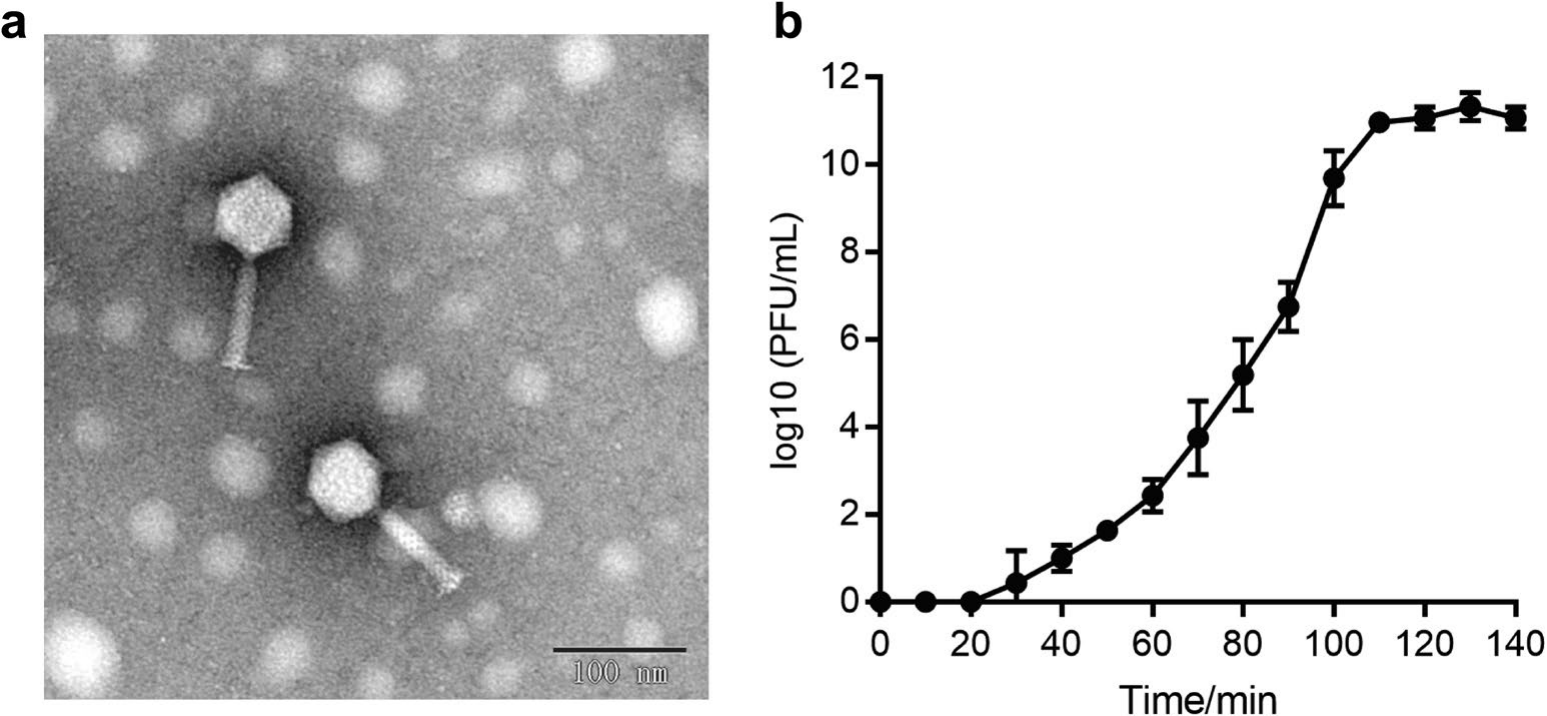
**a** Transmission electron microscopy of phage vB_KpnM_IME346. Scale bar indicates 100 nm; **b** one-step growth curve of phage vB_KpnM_IME346. The phage vB_KpnM_IME346 showed a 15-min latent period at 37 °C, with an average burst size of 139 phage particles per infected cell after 100 min. The data are expressed as the means ± SD. The curve represents average results from three independent experiments

Using *K. pneumoniae* KP576 as an indicator strain, a previously unidentified phage was isolated and designated as vB_KpnM_IME346. Electron micrography showed that the phage had a typical icosahedral structure and contractile tail, with a head diameter of approximately 53 ± 1 nm and tail length of approximately 83 ± 2 nm, which are characteristic features of phages into the family *Myoviridae* (Fig. 1a).

### Biological Properties of Phage vB_KpnM_IME346

The one-step growth curve of the phage showed an incubation period of approximately 20 min. During this period, the number of plaques did not increase, indicating that the phage had not completed replication and assembly. After reaching the plateau phase at 110 min, the burst size was approximately 25–30 plaque-forming unit/cell, indicating that the phage had completely lysed the host cells. The burst size was calculated as the ratio of the final count of phage particles released at the end of the incubation period to the initial count of infected bacterial cells (Fig. 1b).

### Host Range Determination

Twelve *K. pneumoniae* (including capsular type K63 (3/12), K47 (4/12), KN3 (1/12), K64 (2/12), and K81 (2/12)) strains were used for phage lysis assays to determine the lytic host range of phage vB_KpnM_IME346. Notably, phage vB_KpnM_IME346 lysed 3 K63 strains but had no effect on strains with capsular-type K47, KN3, K64, and K81 (Table 1). The phage specifically targeting *K. pneumoniae* capsular-type K63 strains showed specific host ranges, indicating that it may be useful typing of *K. pneumoniae*.

### Genomic Annotation and Analysis

**Fig. 2:**
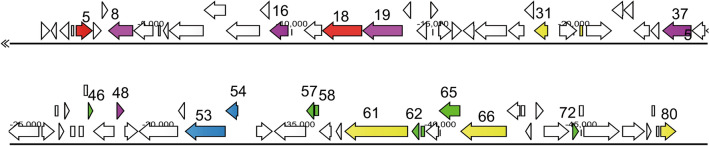
Phylogenetic relationships of phage vB_KpnM_IME346 and other phages belonging to the family *Myoviridae* based on the DNA polymerase amino acid sequences using MEGA6. The phylogenetic tree was generated using the neighbor-joining method and 1000 bootstrap replicates. Nucleotide sequences were not compared. The triangle highlights the phage vB_KpnM_IME346

The whole-genome sequence of phage vB_KpnM_IME346 has been deposited in the NCBI database (GenBank accession number: MK685667). The Phage termini of vB_KpnM_IME346 were identified using our proposed “terminus analysis theory” method, without identification of any fixed termini [23]. Therefore, phage vB_KpnM_IME346 genome may adopt the rolling-circle replication mechanism to generate a number of head-to-tail DNA concatemers which serve as substrates for viral DNA packaging [24]. The assembled phage vB_KpnM_IME346 has a linear double-stranded DNA genome with a G+C content of 49.1%. A total of 80 putative open reading frames (ORFs) were predicted in the phage genome, with ATG as the start codon for 77 ORFs and GAT, GTG, and TTG as the start codons for the remaining three ORFs. Putative functions were assigned to 32 products of predicted vB_KpnM_IME346 ORFs, and the remaining 48 ORFs encoded putative hypothetical proteins. No tRNA genes were identified. The entire genome structure of phage vB_KpnM_IME346 is shown in Fig. 2; the arrows represent predicted ORFs consisting of genes involved in the phage structure, metabolism, phage structure, DNA replication and regulation, and in the lysis system as well as other predicted functions (Table 1).

**Table 1:** Host range of phage vB_KpnM_IME346

Number	Strain	Capsular type	EOP
Host strain	*K. pneumoniae* 576	K63	1
1	*K. pneumoniae* 465	K63	0.78
2	*K. pneumoniae* 501	K63	1.02
3	*K. pneumoniae* 1165	K63	0.99
4	*K. pneumoniae* 1171	K47	–
5	*K. pneumoniae* 2236	K47	–
6	*K. pneumoniae* 2353	K47	–
7	*K. pneumoniae* 2407	K47	–
8	*K. pneumoniae* 2395	K64	–
9	*K. pneumoniae* 2685	K64	–
10	*K. pneumoniae* 1146	KN3	–
11	*K. pneumoniae* 1556	K81	–
12	*K. pneumoniae* 2068	K81	–

“−” indicates that no plaques were observed

**Fig. 3:**
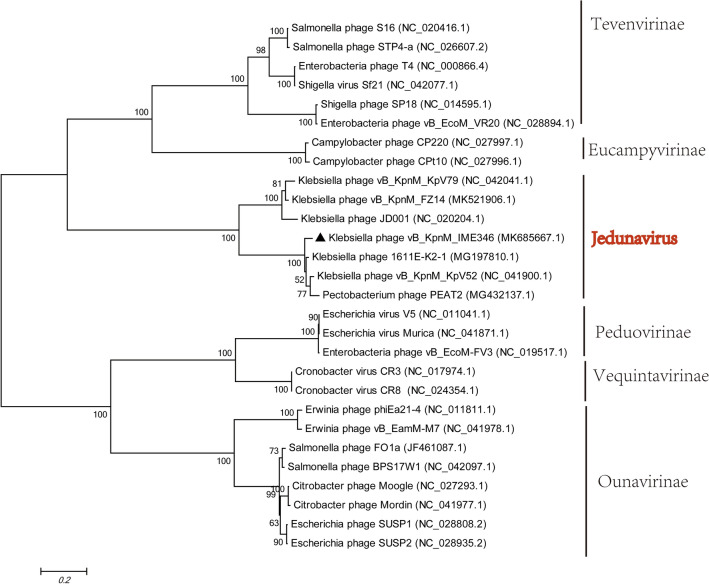
Phage vB_KpnM_IME346 genome map. Arrows represent predicted genes, and the direction of each arrow represents the direction of transcription. Different colors denote different phage gene functional groups. The genome starts upstream of ORF1. The ORF involved in DNA replication and regulation is shown in yellow. The ORF involved in DNA packaging is shown in yellow. The ORF involved in metabolism is shown in green. The ORF involved in structure is shown in purple. Red represents the ORF for phage lytic, and ORFs without phage-related functions are shown in dark red. (Color figure online)

We identified three ORFs as potential genes in the replication and regulation modules, including DNA primase (ORF61), DNA helicase (ORF66), and DNA polymerase (ORF80), which play important roles in phage replication. The metabolism cassette module of the genome contains five ORFs; BLASTn analysis of these regions revealed putative glycosyltransferase (ORF46, ORF58), transketolase protein (ORF57), site-specific DNA-methyltransferase (ORF65), and *N*-acetyltransferase (ORF72), which showed low identity to the corresponding phage sequences in other phages (Fig. 3; Table S1).

Among the 80 predicted ORFs in the phage vB_KpnM_IME346 genome, we identified two putative ORFs encoding proteins associated with lysis function, including endolysin (ORF5) and lytic transglycosylase (ORF18), with a lysozyme-like domain involved in the hydrolysis of beta-1,4-linked polysaccharides. Proteins involved in DNA packaging were encoded by genes in the phage vB_KpnM_IME346 genome, including phage large subunit terminase (ORF53) and a phage small subunit terminase (ORF54), showing 478/482 (99%) and 151/152 (99%) identity to vB_KpnM_KpV79 (NC_042041.1) and JD001 (JX866719.1), respectively. These genes code for the subunits of the terminase protein which is involved in packaging the DNA into the head shell [25, 26]. Five genes were identified to be involved in forming the phage structure. Baseplate protein (ORF16) plays an important role in the infection by phage by catalyzing local cell wall digestion to facilitate penetration of the tail tube through the cell envelope [27]. ORF19 (tape measure protein) was found to be highly similar to that of *Klebsiella* phage JD001, with an identity of 415/477 (87%) and *E*-value of 0. The tape measure protein dictates the tail length and facilitates DNA transit to the cell cytoplasm during infection [28]. Interestingly, a recent study showed that a few *Lactococcus lactis* phages were highly thermoresistant and these results indicate that the tape measure protein contributes to heat stability [29]. ORF48 encoded head morphogenesis protein that is highly similar to *Pectobacterium* phage PEAT2 (MG432137.1) with an identity of 97% (245/253), which mainly mediates phage head assembly.

Additionally, 49 genes of unknown function were identified. Furthermore, no toxin-, virulence factor-, or antibiotic resistance-related genes were found, indicating that vB_KpnM_IME346 is a virulent and potential candidate for phage therapy. The genome of phage vB_KpnM_IME346 contains numerous hypothesis proteins of unknown function; therefore, further comprehensive functional analysis is required to determine the safety of using these phages in therapeutic applications.

BLAST alignment (megablast) indicated that the genome of phage vB_KpnM_IME346 showed the greatest nucleotide sequence identity (90.36% identity, 66% coverage) with the genome sequence of *Klebsiella* phage vB_KpnM_KpV52 (NC_041900.1). CoreGenes 3.5 analysis revealed that 55 genes were shared by vB_KpnM_IME346 and vB_KpnM_KpV52, whereas 24 genes were unique to vB_KpnM_IME346. Among the unique genes, the main functional proteins were glycosyltransferase (ORF58), site-specific DNA-methyltransferase (ORF65), *N*-acetyltransferase (ORF72), and large subunit terminase (ORF53).

To investigate the taxonomy of phage vB_KpnM_IME346, a phylogenetic tree was constructed using the DNA polymerase sequences, including that of the phage and those of other phages in the genus *Jedunavirus*. The results obtained from the phylogenetic analysis that phage vB_KpnM_IME346 belongs to a JD001-like virus of unclassified genus *Jedunavirus* in the family *Myoviridae* (Fig. 3). Given the morphology, phylogenetic relatedness, and complete genome sequence of phage vB_KpnM_IME346 (low query cover to six known JD001-like viruses), these findings indicate that phage vB_KpnM_IME346 is a JD001-like virus in the genus *Jedunavirus* in the family *Myoviridae*. However, further studies evaluating multiple genes in the phage vB_KpnM_IME346 genome are needed to confirm this result [30].

We investigated the biological properties and genomic organization of virulent bacteriophage vB_KpnM_IME346, which infected the clinical *K. pneumoniae* KP576 strain. Importantly, toxin-, pathogen-, and drug resistance-related genes were not detected in the vB_KpnM_IME346 genome. Overall, the good lytic ability and unique genomic characteristics of vB_KpnM_IME346 highlight its potential for use as a clinical phage capsule type and drug-resistant treatment for *K. pneumoniae*.

## Supplementary Information

Below is the link to the electronic supplementary material.

Supplementary file1 (DOCX 27 KB)
