# Supplementary material for: Characterization and Genomic Analysis of Bacteriophage vB_KpnM_IME346 Targeting Clinical Klebsiella pneumoniae Strain of the K63 Capsular Type
Source: Curr Microbiol. 2022 Apr 13;79(6):160. doi: 10.1007/s00284-022-02834-4 (PMC9007800; doi:10.1007/s00284-022-02834-4)
Supplement: Supplementary file 1 — Supplementary file1 (DOCX 27 KB) [file 284_2022_2834_MOESM1_ESM.docx]

Table S1 ORF analysis of the IME346 genome

| ORF | Top BLAST hit a |  | start | stop | % identity ^b^ (aa) | *E* value^c^ |
| --- | --- | --- | --- | --- | --- | --- |
| 1 | hypothetical protein [Atlantibacter subterranea] | ATG | 1106 | 1435 | 42/109(39%) | 6.00E-18 |
| 2 | hypothetical protein [Klebsiella pneumoniae] | ATG | 1667 | 1452 | 71/71(100%) | 4.00E-43 |
| 3 | hypothetical protein [Klebsiella pneumoniae] | GAG | 2110 | 1760 | 114/116(98%) | 2.00E-78 |
| 4 | hypothetical protein [Klebsiella phage vB_KpnM_KpV79] | ATG | 2284 | 2174 | 35/36(97%) | 3.00E-14 |
| 5 | endolysin [Klebsiella phage vB_KpnM_KpV79] | ATG | 2339 | 2944 | 183/186(98%) | 3.00E-133 |
| 6 | putative inner membrane spanin subunit [Edwardsiella phage PEi21] | ATG | 2946 | 3260 | 31/105(30%) | 4.00E-06 |
| 7 | putative outer membrane spanin subunit [Edwardsiella phage PEi21] | ATG | 3257 | 3490 | 33/79(42%) | 1.00E-11 |
| 8 | putative tail-fiber protein [Klebsiella phage 1611E-K2-1] | ATG | 4378 | 3485 | 283/297(95%) | 5.00E-168 |
| 9 | DUF2612 domain-containing protein [Klebsiella pneumoniae] | ATG | 5095 | 4382 | 217/237(92%) | 4.00E-161 |
| 10 | hypothetical protein [Klebsiella quasipneumoniae] | ATG | 5352 | 5254 | 26/29(90%) | 3.00E-09 |
| 11 | hypothetical protein [Klebsiella pneumoniae] | ATG | 5633 | 5427 | 66/67(99%) | 4.00E-42 |
| 12 | hypothetical protein [Escherichia coli] | ATG | 6876 | 5641 | 400/411(97%) | 0.00E+00 |
| 13 | hypothetical protein [Klebsiella pneumoniae] | ATG | 7665 | 6877 | 165/190(87%) | 4.00E-116 |
| 14 | hypothetical protein [Klebsiella pneumoniae] | ATG | 8870 | 7662 | 394/402(98%) | 0.00E+00 |
| 15 | hypothetical protein [Klebsiella phage JD001] | ATG | 9210 | 8863 | 114/115(99%) | 2.00E-75 |
| 16 | putative baseplate protein [Klebsiella phage vB_KpnM_KpV79] | ATG | 9884 | 9210 | 222/224(99%) | 4.00E-163 |
| 17 | hypothetical protein [Pectobacterium phage PEAT2] | ATG | 11077 | 10427 | 214/216(99%) | 4.00E-152 |
| 18 | lytic transglycosylase [Pectobacterium phage PEAT2] | ATG | 12498 | 11074 | 452/474(95%) | 0.00E+00 |
| 19 | tape measure protein [Klebsiella phage JD001] | ATG | 13934 | 12501 | 415/477(87%) | 0.00E+00 |
| 20 | hypothetical protein [Klebsiella phage JD001] | ATG | 14230 | 13934 | 97/98(99%) | 4.00E-66 |
| 21 | hypothetical protein [Klebsiella pneumoniae] | ATG | 14791 | 14417 | 123/124(99%) | 2.00E-84 |
| 22 | hypothetical protein [Klebsiella pneumoniae] | ATG | 14910 | 15194 | 91/94(97%) | 2.00E-62 |
| 23 | hypothetical protein SeSz2_27 [Salmonella phage SeSz-2] | ATG | 15191 | 15661 | 59/157(38%) | 2.00E-25 |
| 24 | hypothetical protein [Klebsiella pneumoniae] | ATG | 15668 | 16033 | 60/80(75%) | 6.00E-36 |
| 25 | hypothetical protein [Klebsiella pneumoniae] | ATG | 16476 | 16060 | 137/138(99%) | 2.00E-93 |
| 26 | hypothetical protein [Pectobacterium phage PEAT2] | ATG | 17626 | 16490 | 366/378(97%) | 0.00E+00 |
| 27 | hypothetical protein [Klebsiella pneumoniae] | ATG | 18248 | 17667 | 188/193(97%) | 3.00E-139 |
| 28 | hypothetical protein [Klebsiella phage JD001] | ATG | 18588 | 18235 | 109/117(93%) | 1.00E-76 |
| 29 | hypothetical protein [Klebsiella phage JD001] | ATG | 19088 | 18585 | 141/167(84%) | 2.00E-98 |
| 30 | hypothetical protein [Escherichia coli] | GTG | 19479 | 20117 | 26/67(39%) | 3.00E-06 |
| 31 | putative iron-only hydrogenase system regulator [Klebsiella phage JD001] | ATG | 20197 | 20328 | 36/43(84%) | 2.00E-17 |
| 32 | hypothetical protein NVP1024O_26 [Vibrio phage 1.024.O._10N.261.45.F8] | ATG | 20431 | 21348 | 90/274(33%) | 2.00E-39 |
| 33 | DUF4054 domain-containing protein [Klebsiella pneumoniae] | ATG | 21756 | 21337 | 87/139(63%) | 2.00E-60 |
| 34 | hypothetical protein [Klebsiella pneumoniae] | ATG | 22114 | 21758 | 115/118(97%) | 2.00E-78 |
| 35 | hypothetical protein [Klebsiella phage JD001] | ATG | 22695 | 22111 | 178/194(92%) | 1.00E-125 |
| 36 | hypothetical protein [Klebsiella phage JD001] | ATG | 23031 | 22714 | 99/105(94%) | 4.00E-64 |
| 37 | major capsid protein [Klebsiella phage vB_KpnM_KpV79] | ATG | 24170 | 23136 | 337/344(98%) | 0.00E+00 |
| 38 | hypothetical protein [Klebsiella pneumoniae] | ATG | 24661 | 24173 | 158/162(98%) | 3.00E-103 |
| 39 | DUF2213 domain-containing protein [Klebsiella pneumoniae] | ATG | 25788 | 24673 | 309/371(83%) | 0.00E+00 |
| 40 | DUF3310 domain-containing protein [Klebsiella pneumoniae] | ATG | 25865 | 26338 | 66/72(92%) | 5.00E-39 |
| 41 | hypothetical protein [Klebsiella phage JD001] | ATG | 26335 | 26466 | 40/43(93%) | 3.00E-21 |
| 42 | hypothetical protein SUNLIREN_4 [Erwinia phage SunLIRen] | ATG | 26469 | 26675 | 31/69(45%) | 3.00E-08 |
| 43 | hypothetical protein [Klebsiella pneumoniae] | ATG | 26672 | 26869 | 61/65(94%) | 1.00E-38 |
| 44 | DUF551 domain-containing protein [Hafnia paralvei] | ATG | 26883 | 27065 | 32/59(54%) | 2.00E-13 |
| 45 | hypothetical protein vsip_50 [Salmonella virus VSiP] | ATG | 27184 | 27363 | 26/53(49%) | 5.00E-11 |
| 46 | putative glycosyltransferase [Klebsiella phage vB_KpnM_KpV79] | ATG | 27360 | 27509 | 41/49(84%) | 1.00E-21 |
| 47 | outer membrane adhesin like protein [Klebsiella phage JD001] | ATG | 27506 | 27703 | 59/63(94%) | 2.00E-35 |
| 48 | head morphogenesis protein [Pectobacterium phage PEAT2] | ATG | 28454 | 27693 | 245/253(97%) | 0.00E+00 |
| 49 | hypothetical protein Sf11_gp59 [Shigella phage Sf11 SMD-2017] | ATG | 28523 | 28807 | 49/92(53%) | 6.00E-21 |
| 50 | hypothetical protein A3466_07955 [Enterobacter genomosp. S] | ATG | 28804 | 29298 | 83/163(51%) | 4.00E-45 |
| 51 | DUF1073 domain-containing protein [Klebsiella pneumoniae] | ATG | 30709 | 29312 | 452/464(97%) | 0.00E+00 |
| 52 | DUF2829 domain-containing protein [Enterobacter cloacae] | ATG | 30951 | 30706 | 52/76(68%) | 1.00E-32 |
| 53 | large subunit terminase [Klebsiella phage vB_KpnM_KpV79] | ATG | 32396 | 30948 | 478/482(99%) | 0.00E+00 |
| 54 | phage small subunit terminase [Klebsiella phage JD001] | ATG | 32844 | 32386 | 151/152(99%) | 4.00E-105 |
| 55 | hypothetical protein [Pectobacterium phage PEAT2] | ATG | 33475 | 34062 | 94/193(49%) | 5.00E-64 |
| 56 | hypothetical protein [Klebsiella phage JD001] | ATG | 35249 | 34107 | 372/380(98%) | 0.00E+00 |
| 57 | transketolase protein [Pectobacterium phage PEAT2] | ATG | 35539 | 35249 | 89/96(93%) | 2.00E-59 |
| 58 | putative glycosyltransferase [Klebsiella phage JD001] | ATG | 35705 | 35532 | 55/99(56%) | 3.00E-24 |
| 59 | hypothetical protein [Klebsiella pneumoniae] | ATG | 36151 | 35702 | 66/119(55%) | 1.00E-27 |
| 60 | hypothetical protein kpv52_69 [Klebsiella phage vB_KpnM_KpV52] | ATG | 36509 | 36294 | 65/71(92%) | 5.00E-39 |
| 61 | putative DNA primase [Klebsiella phage vB_KpnM_KpV52] | ATG | 38863 | 36605 | 725/752(96%) | 0.00E+00 |
| 62 | TetR family transcriptional regulator [Klebsiella pneumoniae] | ATG | 39263 | 38985 | 89/92(97%) | 1.00E-57 |
| 63 | no hits | TTG | 39306 | 39461 | 0 | 0 |
| 64 | hypothetical protein [Pectobacterium phage PEAT2] | ATG | 39955 | 39482 | 146/157(93%) | 3.00E-103 |
| 65 | site-specific DNA-methyltransferase [Moraxella porci] | ATG | 40716 | 39952 | 158/240(66%) | 3.00E-118 |
| 66 | DNA helicase [Klebsiella phage vB_KpnM_KpV79] | ATG | 42362 | 40716 | 366/543(67%) | 0.00E+00 |
| 67 | DUF1273 family protein [Massilia alkalitolerans] | ATG | 42841 | 42359 | 81/154(53%) | 1.00E-50 |
| 68 | hypothetical protein kpv52_61 [Klebsiella phage vB_KpnM_KpV52] | ATG | 43015 | 42854 | 49/53(92%) | 5.00E-27 |
| 69 | hypothetical protein [Klebsiella pneumoniae] | ATG | 43239 | 43012 | 57/75(76%) | 1.00E-36 |
| 70 | hypothetical protein phiTE_188 [Pectobacterium phage phiTE] | ATG | 43381 | 43671 | 51/93(55%) | 3.00E-24 |
| 71 | hypothetical protein [Klebsiella phage JD001] | ATG | 43668 | 44579 | 231/316(73%) | 6.00E-128 |
| 72 | N-acetyltransferase [Aliihoeflea sp. 2WW] | ATG | 44684 | 44923 | 17/59(29%) | 9.6 |
| 73 | no hits | ATG | 44920 | 45090 | 0 | 0 |
| 74 | DUF2800 domain-containing protein [Klebsiella pneumoniae] | ATG | 45075 | 46364 | 360/373(97%) | 0.00E+00 |
| 75 | hypothetical protein [Klebsiella pneumoniae] | ATG | 46451 | 47260 | 263/270(97%) | 0.00E+00 |
| 76 | hypothetical protein GAP31_015 [Cronobacter phage vB_CsaM_GAP31] | ATG | 47309 | 47497 | 31/58(53%) | 7.00E-12 |
| 77 | hypothetical protein [Klebsiella phage JD001] | ATG | 47494 | 47619 | 38/38(100%) | 7.00E-18 |
| 78 | DUF2158 domain-containing protein [Klebsiella pneumoniae] | ATG | 47655 | 47774 | 33/36(92%) | 7.00E-15 |
| 79 | hypothetical protein [Mesorhizobium sp. M1A.F.Ca.ET.072.01.1.1] | ATG | 47804 | 48373 | 61/168(36%) | 9.00E-26 |
| 80 | DNA polymerase [Pectobacterium phage PEAT2] | ATG | 48370 | 1065 | 329/371(89%) | 0.00E+00 |

a the most closely related protein and its organism. ‘‘No hits’’ indicates no significant hits detected for a particular amino acid sequence

b percent identity for top hits in BLASTP searches. Numbers in parentheses provide length of each alignment

c the probability of obtaining a match by chance as determined by BLASTP analysis
